# Prevalence of stroke in Pakistan: Findings from Khyber Pakhtunkhwa integrated population health survey (KP-IPHS) 2016-17

**DOI:** 10.12669/pjms.36.7.2824

**Published:** 2020

**Authors:** Akhtar Sherin, Zia Ul-Haq, Sheraz Fazid, Basharat Hussain Shah, Maria Ishaq Khattak, Fazal Nabi

**Affiliations:** 1 Akhtar Sherin Khyber Medical University, Institute of Medical Sciences, DHQ Hospital Kohat, Pakistan. Khyber Medical University, Institute of Public Health & Social Sciences, Peshawar, Pakistan; 2 Zia Ul-Haq, Vice Chancellor/ Professor & Dean, Institute of Public Health. Visiting Professor Institute of Health & Well-being, University of Glasgow, UK. Khyber Medical University, Institute of Public Health & Social Sciences, Peshawar, Pakistan; 3 Sheraz Fazid Institute of Public Health, KMU Khyber Medical University, Institute of Public Health & Social Sciences, Peshawar, Pakistan; 4 Basharat Hussain Shah Khyber Medical University, Institute of Public Health & Social Sciences, Peshawar, Pakistan; 5 Maria Ishaq Khattak Lecturer, Institute of Public Health, KMU Khyber Medical University, Institute of Public Health & Social Sciences, Peshawar, Pakistan; 6 Fazal Nabi Population Welfare Department, Government of Khyber Pakhtunkhwa, Peshawar, Pakistan

**Keywords:** KP-IPHS, Stroke, Prevalence, Risk factors, Hypertension, Diabetes Mellitus, Obesity, Smoking, Dyslipidemias, Pakistan

## Abstract

**Objective:**

To study the prevalence of stroke and associated risk factors in Khyber Pakhtunkhwa (KP) province of Pakistan.

**Methods:**

This study was a part of cross-sectional KP Integrated Population Health Survey 2016-17 conducted on population aging ≥18 years at 24 districts of KP. Primary (n=1061) and secondary sampling units (n=15724) were developed, based on urban/rural and socio-economic status. Each primary-unit comprised of 250-300 households. Sample was selected through a multi-staged stratified systematic cluster sampling technique by taking every 16^th^ household per rural and every 12^th^ household per urban-unit. A validated “*Cincinnati Stroke Scale”* for identification of stroke patients in community was used along with demographics and potential risk factors.

**Results:**

Among the 15724 randomly selected households, 22500 participants (51.4% females; 74.6% rural areas, mean age 42±12.6 years) were interviewed. Stroke was identified in 271 cases (137 males, 134 females; Mean age=43.39±0.85 years) and prevalence of stroke was 1.2% (1200 per 100,000 population). Obesity/overweight (38.8%), hypertension (21.8%), smoking (6.6%) and known diabetes mellitus (5.9%) were the common associated risk factors of stroke. Age groups >60 years (adjusted OR=1.68; 95% CI: 1.05-2.68); urban area (adjusted OR=1.68; 95% CI: 1.29-2.19); unemployment (adjusted OR=3.78; 95% CI: 2.49-5.73) and lower formal (primary) education (adjusted OR 2.18; 95% CI: 1.30-3.64) were significantly associated with stroke (p <0.05).

**Conclusion:**

Prevalence of stroke is 1.2% in the province of KP. Obesity, hypertension, smoking and Diabetes Mellitus are the common associated risk factors of stroke. Higher age, urban area, unemployment and lower formal education are significantly associated with stroke.

## INTRODUCTION

Stroke is the second leading cause of global mortality after ischemic heart disease, accounting for about 10.2% of global deaths in 2016.^1^ Stroke contributed for 67.3% of deaths due to all neurological disorders.^2^ Stroke was also responsible for 5.2% of global disability-adjusted life years (DALYs) lost in 2016.^1^ Worldwide, about 15 million new people develop a stroke on annual basis.^3^ Prevalence of stroke is increasing over the last three decades with annual increase of 14.3% being reported in low-incomes countries.^4^ Significant variations in prevalence, morbidity and mortality of stroke have been reported across different geographic l ocations and regions, especially in countries with different socioeconomic status.^4^-^6^

South Asia, a major contributing region of the global stroke burden, showed gross difference in prevalence rates of stroke across different countries.^7^ Stroke in South Asia is different from rest of the world, in terms of higher prevalence rate, younger age, high mortality, increased burden of modifiable risk factors of stroke and some under-researched non-conventional risk factors.^7^-^9^

Pakistan, currently the sixth most populous country of the world, has limited population-based, robust data on prevalence of stroke and its risk factors. Only two small-scale population-based studies were conducted, a decade ago in Karachi, the single metropolitan city of Pakistan. Prevalence of stroke in these studies was 4.8%^10^ & 19.1%,^11^ highest in the region. A large scale, population-based was needed to study the prevalence of stroke in rural and urban areas of Pakistan. This study was planned to find out the prevalence of stroke and its common modifiable risk factors in adult people living in Khyber Pakhtunkhwa province of Pakistan.

## METHODS

This cross-sectional study was a part of Khyber Pakhtunkhwa Integrated Population and Health Survey (KP-IPHS) 2016-17,^12^ to collect comprehensive information about demographic characteristics and locale-specific health-related issues of people of the KP, Pakistan. Sample size (n=15724 households) was estimated by Bureau of statistics planning & development department government of KP (http://kpbos.gov.pk/). Study was conducted during 2016-17 on adult population of either gender with age of 18 years or more, living in rural and urban areas of 24 district of KP province of Pakistan. Informed consent was taken from all participants.

A multi-staged stratified cluster sampling technique was used for the selection of sample. Sampling included all districts of Khyber Pakhtunkhwa comprising of strata from *rural/urban* and *low-, middle- and high-income* groups. *Urban areas* were further divided into *enumeration blocks* based on the *low-, middle- and high-income groups*, while *rural areas* were divided into *mohalla/villages*. These were the *primary sampling units* (PSU’s). Each PSU comprised of 250-300 households. Listing was done for selected enumeration blocks for urban and mohallas/villages for rural areas and all households in each PSU. *Secondary sampling units* (SSU’s) were selected for data collection through *systematic random sampling* by taking every 16^th^ household per rural PSU and every 12^th^ household per urban PSU. A total of 3756 households were selected from the urban areas of the seven districts and 11968 households were selected from the rural areas of these 24 districts. The total sample size of the study was 15724 households.

We used *Cincinnati Stroke Scale* for identification of stroke patients in community.^13^ This simple scale, was initially designed for out-of-hospital identification of stroke patients by physician and later on was validated for detection of patients with stroke by prehospital care providers^14^ as well as by even laypersons and showed an overall diagnostic accuracy of 98%, sensitivity of 91% and specificity of 88%.^15^

This scale was incorporated in KPIPHS questionnaire, designed to collect multi-dimensional information regarding various aspects of people of KP, Pakistan. We also recorded the data regarding demographic details, co-morbid conditions, body mass index (BMI), waist to hip ratio (WHR), physical activity, common modifiable risk factors of stroke including hypertension, diabetes, angina / ischemic heart disease, smoking and obesity. Data collection was done through priorly trained field workers (30-40 per district) and data monitoring was done by district demographers of Population Welfare Department of KP. Data was analyzed through STATA software. Ethical approval of the study was taken from the ethical committee of the Khyber Medical University (KMU), Peshawar, Pakistan.

## RESULTS

Out of 22500 participants of study, 11,556 (51.4%) were females and 10,944 (48.6%) were males. Mean age of the study participants was 42±12.6 years. About 66.4% (n=14942) of our study population was less than 50 years of age. Majority (74.66%) were from rural areas and about 10.9% had no formal education.

Stroke was diagnosed in 271/22500 cases and prevalence of stroke was 1.2% (1200 per 100,000 population). Male-female ratio in stroke patients was 1.02:1. Prevalence of stroke in males & female population was 1.3% (n=137/10944) & 1.2% (n=134/11556) respectively. Mean age of stroke patients was 43.39±0.85 (95% CI: 41.71-45.07) years. About 66.8% (n=181) of stroke patients were younger than 50 years of age and 16.97% (n=46) were aging more than 60 years (Table-I). Prevalence of stroke in individuals less than 50 years of age was 1.2% and in those, having age more than 60 years was 2.03%.

**Table-I:** Characteristics of the study participants using chi-square statistics (n=22500).

Variable		Stroke Positive n (%) 271 (1.2)	Stroke negative n (%) 22,229 (98.8)	p-value (Chi Square)
Age Group (years)	<30	37 (13.65)	3,599 (16.19)
	30-39	77 (28.41)	5,872 (26.42)
	40-49	67 (24.72)	5,290 (23.8)
	50-59	44 (16.24)	5,247 (23.61)
	60 & above	46 (16.97)	2,219 (9.98)
Gender	Male	137 (50.55)	10,806 (48.62)	0.52
	Female	134 (49.45)	11,421 (51.38)	
Area	Urban	90 (33.21)	5,611 (25.24)	0.003
	Rural	181(66.79)	16,618 (74.76)	
Employment status	Employed	129 (47.6)	11,099 (49.93)
	Unemployed	74 (27.31)	3,546 (15.95)
	Housewife	68 (25.09)	7,584 (34.12)
Years of education	No formal education	18 (6.64)	2,425 (10.91)
	Primary	168 (61.99)	12,630 (56.82)
	Secondary	60 (22.14)	4,548 (20.46)
	Graduate	25 (9.23)	2,624 (11.81)
Body Mass Index	Underweight	33 (12.18)	1,471 (6.62)
	Normal weight	133 (49.08)	12,026 (54.1)
	Over weight	69 (25.46)	4,846 (21.8)
	Obese class 1	28 (10.33)	2,434 (10.95)
	Obese class 2	5 (1.85)	850 (3.82)
	Obese class 3	3 (1.11)	602 (2.71)
Hypertension	Yes	59 (21.77)	4,199 (18.89)	0.229
	No	212 (78.23)	18,028 (81.11)	
Diabetes	Yes	16 (5.9)	843 (3.79)	0.07
	No	255 (94.1)	21,386 (96.21)	
Smoking	Yes	18 (6.64)	1,755 (7.9)	0.447
	No	253 (93.36)	20,474 (92.1)	

About 66.8% (n=181/271) of stroke patients were settled in rural areas Prevalence of stroke in urban and rural areas was 1.58% (n=90/5701) & 1.1% (n=181/16799) respectively. Majority (62%) of stroke patients had some formal education at primary level.Hypertension (21.77%), smoking (6.64%), Diabetes Mellitus (5.9%), Dyslipidemia (3.32%), history of myocardial infarction (3.32%) and angina (2.21%) were the common risk factors of stroke. Prevalence of stroke in diabetic, hypertensive patients & smokers was 1.9%, 1.4% & 1.02% respectively.

About 25.5% (n=69/271) of stroke patients were overweight & 13.2% (n=36) were obese (as per Asian cutoff score for obesity). Prevalence of stroke in overweight or obese people was 1.2% (n=105/8837). Mean BMI of stroke patients was 22.75 (95% CI: 22.15-23.34). Data for WHR was available for only 6,695 (31.3%) of study population including 101 subjects with stroke. Out of these 101 stroke patients, WHR was higher than normal in 87.1% (n=88) of cases.

Multivariate regression analysis of various variables like age-category, gender, settlement area, employment, education status, BMI and associated risk factors in patients with stroke is given in Table-II. Age groups >60 years (adjusted OR=1.68; 95% CI: 1.05-2.68); urban area (adjusted OR=1.68; 95% CI: 1.29-2.19); unemployment (adjusted OR=3.78; 95% CI: 2.49-5.73) and lower formal (primary) education (adjusted OR 2.18; 95% CI: 1.30-3.64) were significantly associated with stroke (p <0.05).

**Table-II:** Logistic regression of participants characteristic associated with having stroke (n=22500).

Variables		Unadjusted results			Adjusted results		

		OR	95% CI	p-value	OR	95% CI	p-value
Age (Years)	<30	*Ref*			*Ref*		
	30-39	1.28	0.86, 1.89	0.226	1.31	0.88, 1.95	0.182
	40-49	1.23	0.82, 1.84	0.311	1.26	0.84, 1.91	0.268
	50-59	0.82	0.53, 1.27	0.363	0.66	0.41, 1.04	0.075
	60 & above	2.02	1.30, 3.12	0.002	1.68	1.05, 2.68	0.029
Gender	Male	*Ref*			*Ref*		
	Female	0.93	0.73, 1.18	0.526	1.70	1.18, 2.45	0.005
Residence area	Rural	*Ref*			*Ref*		
	Urban	1.47	1.14, 1.89	0.003	1.68	1.29, 2.19	<0.001
employment status	Housewife	*Ref*			*Ref*		
	Employed	1.29	0.96, 1.74	0.085	2.26	1.56, 3.26	<0.001
	Unemployed	2.32	1.67, 3.24	<0.001	3.78	2.49, 5.73	0.001
Education status	No formal education	*Ref*			*Ref*		
	Primary	1.79	1.10, 2.92	0.019	2.18	1.30, 3.64	0.003
	Secondary	1.78	1.05, 3.02	0.033	1.71	1.00, 2.91	0.048
	Graduation	1.28	0.70, 2.36	0.421	1.20	0.65, 2.23	0.554
Body mass index	Normal weight	*Ref*			*Ref*		
	Underweight	2.03	1.38, 2.98	<0.001	1.91	1.29, 2.83	0.001
	Overweight	1.29	0.96, 1.73	0.091	1.29	0.95, 1.74	0.099
	Obese class1	1.04	0.69, 1.57	0.851	1.02	0.67, 1.54	0.933
	Obese class 2	0.53	0.22, 1.30	0.167	0.51	0.21, 1.25	0.141
	Obese class 3	0.45	0.14, 1.42	0.173	0.44	0.14, 1.39	0.161
Hypertension	Non-hypertensive	*Ref*			*Ref*		
	Hypertensive	0.84	0.63, 1.12	0.229	0.88	0.65, 1.19	0.397
Diabetes	Non-diabetic	*Ref*			*Ref*		
	Diabetic	0.63	0.38, 1.05	0.074	0.65	0.39, 1.09	0.104
Smoking	Non-smoker	*Ref*			*Ref*		
	Smoker	1.20	0.75, 1.95	0.447	1.24	0.76, 2.03	0.385

## DISCUSSION

In this study, prevalence of stroke in KP province of Pakistan was 1.2% (1200 per 100,000 population). This figure is quite different from two previously conducted population based studies in Pakistan, showing 4.8% (4800 per 100,000 population)^10^ & 19.1% (19100 per 100,000 population)^11^ prevalence of stroke in Pakistan. Both these studies were conducted in city of Karachi and findings cannot be generalized to entire country. Jafar TH^10^ conducted study on 500 subjects of Pashtun ethnic population of Karachi and used a non-validated questionnaire for screening of stroke patients. Kamal AK^11^ used a validated stroke symptoms questionnaire and neurologist confirmed stroke in screened positive patients. However, the study was conducted only in a small urban slum, including 545 people of low socioeconomic status. The stroke prevalence rate 19% is the highest ever reported from the region and seems to be implausible.

Our findings are more comparable than results of Jafar TH^10^ & Kamal AK^11^, with other studies from South Asian countries. Gupta R et al^16^ reported stroke prevalence of 0.9% in India, Pakistan and Bangladesh. While Prasad K et al^9^ reported prevalence of stroke in 44 to 843 per 100,000 (0.44% to 0.843%) in India. A systematic review and meta-analysis by Chowdhury MZI et al reported a weighted pooled prevalence 1% for stroke In Bangladesh.^17^ In Sri Lanka, stroke prevalence was 1040 per 100,000 (1.04%).^18^ Recently, China reported stroke prevalence of 1115 per 100 000.^19^ Variations in epidemiology of stroke has been reported across the globe as well as in South Asian countries.^20^,^21^ These variations can occur within same country and may represent the actual differences in epidemiology of stroke or showing the heterogeneity in the methodology of the studies or may denote the differences in the time trend.^22^

In our study, age group ≥60 years was significantly associated with risk of stroke in general population. This is in accordance with previously reports that incidence of stroke is increasing with age.^5^ However, there is an increasing trend in incidence of stroke in younger ages.^23^ Stroke at a younger age is more common in South Asia than other regions.^8^ In our study, around two-third of stroke patients were less than fifty years of age. Stroke at a younger age is an alarming situation in Pakistan.^24^ Kamal et al^11^ reported 48.1% & Jafar TH reported 30% of stroke in patient aging forty-five years or less.^10^ Stroke at a younger age could be due to the risk factors like Hypertension, waist-hip ratio, and cardiac risk factors, which are more significantly associated with stroke in people aging ≤55 years than people aging >55 years.^25^

Rural-urban difference in the stroke prevalence is vital for South Asian countries, where almost two third of population is living in rural areas.^7^ This is first population-based study in Pakistan where 74.66% of study sample was proportionately selected from rural areas. The distribution of overall stroke patients in rural areas (66.8%) was accordingly proportionate to sample selection from rural areas. However, multivariate regression analysis showed that settlement in urban area was associated with higher risk of stroke than settlement in rural areas (adjusted OR 1.68). This finding is in conformity with the results of a systematic review by Kulshreshtha A et al. who reported a higher prevalence rate of stroke in urban areas (147–471 per 100,000) as compared to rural areas (45–143 per 100,000) in South Asian countries.^7^

Hypertension, diabetes mellitus, dyslipidemia, smoking, obesity and ischemic heart diseases were the common risk factors of stroke in our population. These findings are in accordance with results of other studies from Pakistan and other South Asian countries.^8^,^10^,^11^,^22^ INTERSTROKE study^25^ showed population attributable risks in patients of South Asian countries for Hypertension (47·2%), Waist-to-hip ratio 32·1%, current smoking (8%), DM (-0·1%) and cardiac causes (5·2%).

### Limitation of the study

This is the largest population-based study on prevalence of stroke in Pakistan and first study including both urban and rural population. However, diagnosis of stroke by healthcare workers was not counter-verified by the physicians, neurologists, or CT/MRI scan.

## CONCLUSION

Prevalence of stroke in KP province of Pakistan is 1.2% (1200 per 100,000 population). Majority of patients were younger than 50 years of age. There is no significant gender-based difference in prevalence of stroke. Obesity, hypertension, smoking and Diabetes Mellitus were the common risk factors of stroke. Higher age, urban area, unemployment and lower formal education were significantly associated with stroke.

### Authors’ Contribution:

**AS:** Conceived idea, data collection, manuscript writing, and final approval of manuscript.

**ZUH:** Study design, data collection, data analysis, manuscript writing, and final approval of manuscript.

**SF & BHS:** Data Analysis & interpretation of data, critical review & and final approval of manuscript.

**MIK & FN:** Supervised data collection, critical review & and final approval of manuscript.
